# Annexin A5 in Ischemic Stroke: Molecular Mechanisms and Translational Therapy Strategies

**DOI:** 10.14336/AD.2025.1021

**Published:** 2026-10-05

**Authors:** Zhanwei Zhu, Jiachen He, Qi Liu, Jiaqi Guo, Jingbei Liu, Shuaili Xu, Xi Chen, Di Wu

**Affiliations:** ^1^Department of Neurology and China-America Institute of Neuroscience, Xuanwu Hospital, Capital Medical University, Beijing 100053, China.; ^2^Beijing Institute of Brain Disorders, Capital Medical University, Beijing 100053, China

**Keywords:** ischemic stroke, annexin a5, neuroprotection, brain, multi-target

## Abstract

Previous research has identified externalized phosphatidylserine (PS) as an "eat me" signal in apoptosis regulation. Targeting PS thus represents a promising therapeutic approach. Annexin A5, known for its high affinity for PS, has traditionally been used as a FITC-labeled probe for detecting apoptosis. Recent studies have demonstrated that Annexin A5 can cross the blood-brain barrier and selectively bind to PS-exposed injured tissues, exerting multiple protective effects, such as anticoagulant, anti-inflammatory, and anti-apoptotic actions. Furthermore, emerging evidence suggests that Annexin A5 holds potential for molecular imaging of the ischemic penumbra and for the development of lesion-targeted drug delivery systems. In summary, Annexin A5 has garnered increasing attention for its neuroprotective properties and therapeutic potential. Ischemic stroke is one of the leading causes of disability and mortality worldwide. Ischemic stroke management still requires effective neuroprotective agents and innovative imaging strategies to improve clinical outcomes. This article provides a comprehensive analysis of protective mechanisms and translational applicability of Annexin A5 in ischemic stroke therapy, offering novel insights for the development of next-generation multi-target cerebrovascular interventions.

## 1. Introduction

Stroke is one of the main causes of permanent disability in adults [1], and ischemic stroke accounts for up to 65% [2]. Currently, intravenous thrombolysis with tissue plasminogen activator (tPA) and mechanical thrombectomy with stent retrievers as the mainstream reperfusion strategies for the treatment of ischemic stroke, have markedly improved patient prognosis [3, 4]. Nevertheless, even after successful recanalization, several challenges persist in the treatment of this condition. These include vascular re-occlusion [5-7], limited drug delivery across the blood-brain barrier (BBB) [8], post-reperfusion coagulopathy [9], hemorrhagic complications following recanalization therapies [10], and the activation of inflammatory cascades in the brain after ischemic stroke [11]. Furthermore, there remains a significant scarcity of effective neuroprotective agents capable of improving long-term functional outcomes [12, 13]. In light of the multifactorial and network-based pathogenesis of brain disorders, conventional neuroprotective drugs targeting singular pathways have yielded suboptimal translational outcomes [14]. Emerging evidence advocates for the development of multitarget agents with broad-spectrum bioactivity, which hold substantial potential to redefine neuroprotective strategies by simultaneously modulating multiple converging pathological cascades [15, 16].

Ischemic penumbra, a hypoperfused yet potentially salvageable region surrounding the infarct core, has long been considered an ideal therapeutic target in ischemic stroke due to its critical role in determining final infarct volume and clinical outcomes [17]. However, the accurate measurement and precise localization of the ischemic penumbra in clinical practice remain highly challenging, owing to limitations in current neuroimaging modalities and the dynamic, heterogeneous nature of cerebral ischemia [17, 18]. This diagnostic uncertainty not only hinders the prompt identification of patients who may benefit from neuroprotective interventions but also contributes to the limited success in translating numerous neuroprotective agents into effective clinical treatments [19]. Moreover, even in cases where neuroprotective drugs exhibit preclinical efficacy, targeted delivery to the lesion site and the maintenance of therapeutic drug concentrations within the penumbral region remain significant challenges in clinical practice. The inability to precisely target and retain therapeutic agents within the penumbra has emerged as an important barrier to optimizing neuroprotective strategies and improving outcomes in ischemic stroke [20, 21].

The Annexin A5 gene was first explored in detail in 1994 [22]. Annexin A5 is expressed in most cells and tissues [23, 24], especially in the lung, placenta, heart, liver, kidney, and multiple vascular endothelial cells [25, 26]. Moreover, Annexin A5 expression is upregulated in response to cell stress or apoptosis [27, 28]. Annexin A5 can enter the circulation through secretion or release [29], and binds PS in a Ca²^+^-dependent manner, mediating multiple functional roles [30], such as anti-inflammatory [31], anticoagulation [32], neuroprotection [33]. As an endogenous molecule in the human body, Annexin A5 is regarded as a potential therapeutic drug to improve the outcome of ischemic stroke [34]. Here, we summarize the research progress of Annexin A5 in ischemic stroke.

## 2. Molecular Pathophysiology of Ischemic Stroke

Ischemic stroke represents a rapidly evolving cerebrovascular event characterized by a sequence of spatially and temporally distinct yet interrelated pathophysiological processes [35]. The onset is precipitated by the abrupt occlusion of a cerebral artery, resulting in immediate cessation of regional cerebral blood flow [36]. This reduction in perfusion rapidly initiates a cascade of biochemical and molecular disturbances within both the vascular compartment and brain parenchyma. One of the earliest responses is the activation of the coagulation system, which is primarily mediated through the coagulation factors. Endothelial injury and exposure of subendothelial matrix components activate platelets and coagulation factors, culminating in the generation of thrombin, fibrin deposition, and subsequent intraluminal thrombus formation [37]. These coagulation-driven events not only perpetuate ischemia by further obstructing residual collateral circulation but also create a highly prothrombotic microenvironment within the ischemic event [38, 39].

Moreover, ischemic injury provokes a robust inflammatory response at the vascular interface [40]. Endothelial cells rapidly upregulate adhesion molecules, facilitating the adhesion and trans-endothelial migration of circulating leukocytes. The neutrophils and monocytes infiltrate the perivascular space and cerebral parenchyma, releasing an array of pro-inflammatory cytokines such as tumor necrosis factor-alpha (TNF-α) and interleukin-6 (IL-6) [41, 42]. These mediators contribute to the destabilization of the BBB [43, 44], amplifying vascular leakage, cerebral edema, and infiltration of peripheral immune cells into the brain parenchyma [45, 46]. Following vascular inflammation, microglia—the brain's intrinsic immune cells—undergo rapid activation within hours after ischemic onset. Initially adopting a pro-inflammatory M1 phenotype, activated microglia produce substantial quantities of reactive oxygen species (ROS), nitric oxide, and pro-inflammatory cytokines, exacerbating local neurotoxicity [47]. Notably, beyond their pro-inflammatory role, the activated microglia phagocytose the surviving but stressed neurons, which will aggravate the secondary neuron loss in the area around the infarction.

Along with these microglia-mediated inflammatory responses, ischemia-induced metabolic failure and excitotoxicity trigger distinct forms of neuronal cell death [48]. In the ischemic core, where cerebral perfusion is critically reduced, neurons rapidly undergo necrosis due to severe ATP depletion, membrane disruption, and irreversible ionic disequilibrium [49]. Necrotic cell death results in the release of the damage-associated molecular patterns (DAMPs) that further potentiate local inflammation; the neurons located within the ischemic penumbra undergo subsequent apoptosis [50]. Apoptosis is mediated through both intrinsic and extrinsic pathways, characterized by mitochondrial outer membrane permeabilization, cytochrome C release, caspase-3 activation, and subsequent DNA fragmentation. These cell death mechanisms are closely related to the occurrence of pathological events such as oxidative stress, mitochondrial dysfunction, and excitotoxic glutamate accumulation, which together amplify the brain damage caused by the ischemic cascade [51] (Fig 1).

In conclusion, coagulation-driven thrombosis, vascular inflammation, phagocytosis of microglia, and successive necrotic and apoptotic neurons are important events in the pathological process of ischemic stroke [52, 53]. Understanding this continuous pathological process is very crucial for finding drugs to alleviate neuronal damage and improve clinical outcomes after ischemic stroke.

## 3. Structural characteristics of Annexin A5

Annexin A5 consists of a highly conserved C-terminal core composed of four homologous domains, each containing approximately 70 amino acid residues, with a short N-terminal domain of about 20 amino acids. This short N-terminal region plays a critical role in regulating the overall conformational stability and modulating the interactions between Domain I and Domain IV on the concave side of the molecule [54-56]. The intramolecular communication from the N-terminal to the loops within Domain III contributes to dynamic conformational flexibility, facilitating membrane interactions and intracellular trafficking [57]. This structural arrangement provides Annexin A5 with the capacity to remain structurally stable while undergoing trimerization, enabling the formation of higher-order assemblies upon membrane binding. These assemblies are essential for its function as a drug carrier, facilitating the formation of membrane invaginations and vesicle-like structures that internalize extracellular materials through a mechanism independent of classical receptor-mediated endocytosis [58].

**Figure 1.**
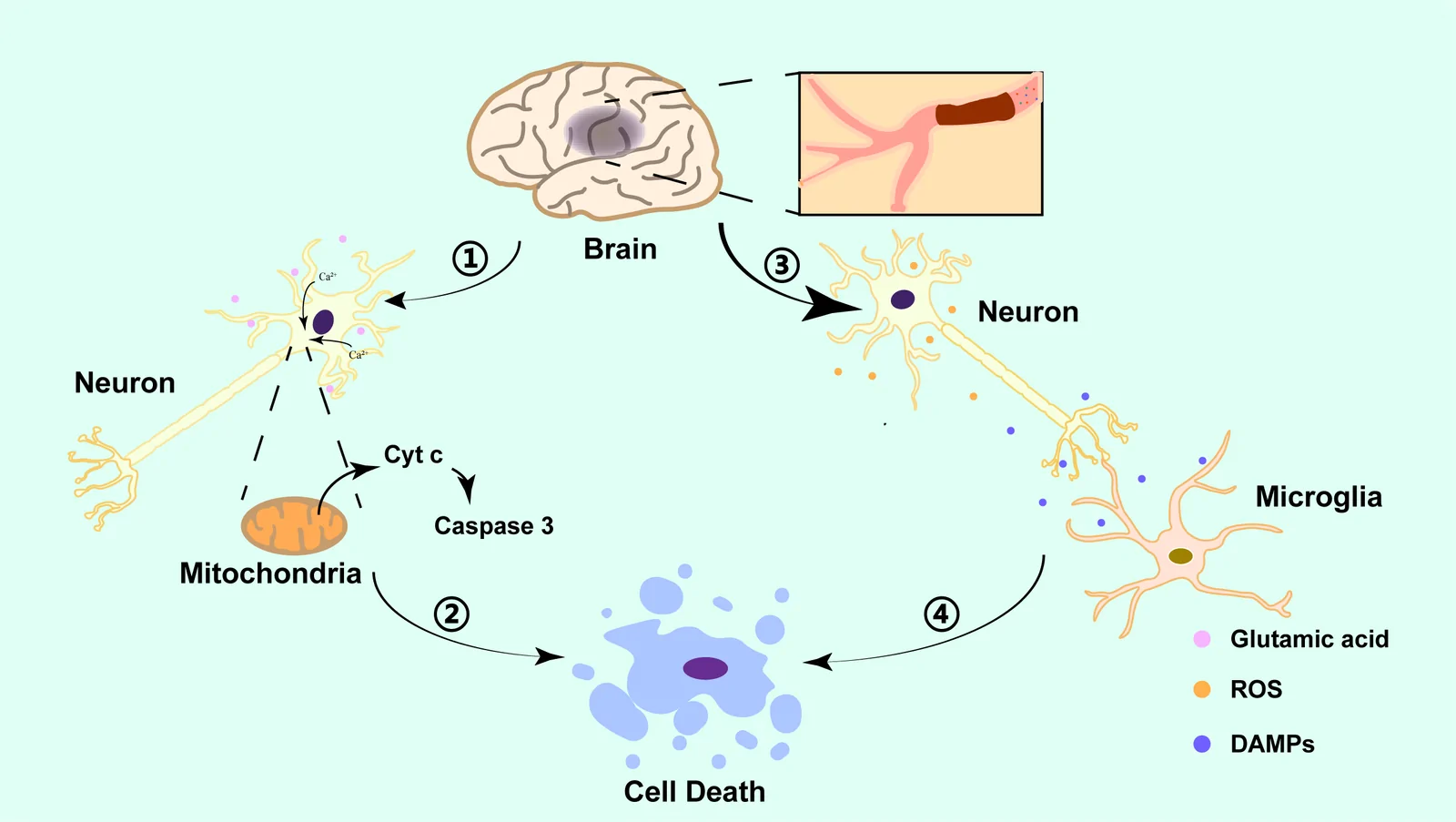
**Pathological processes leading to cell death following the onset of ischemic stroke**. (1) After the onset of cerebral ischemia, excitatory neurotoxicity is initiated, resulting in impaired ion homeostasis in neurons and triggering the apoptotic cascade. (2) Leakage of cytochrome c from mitochondria activates the Caspase-3 apoptotic pathway, ultimately causing cell death. (3) Reperfusion injury may occur following vascular recanalization, during which neurons produce excessive reactive oxygen species (ROS) and damage-associated molecular patterns (DAMPs), thereby recruiting microglia. (4) Microglia subsequently secrete inflammatory cytokines, contributing to further cell death.

Secondly, the overall tertiary structure of Annexin A5 adopts a typical α-type conformation, featuring a disk-like shape composed entirely of α-helices arranged into four domains [59]. The convex surface of this domain harbors five calcium-binding sites, three of which (located in Domains I, II, and IV) exhibit the highest affinity for calcium through strongly liganded interhelical loops containing aspartate (Asp) and glutamate (Glu) residues [55]. This structural arrangement enables Annexin A5 to bind reversibly to negatively charged PS in a calcium-dependent manner, with dissociation constants ranging from 0.1 to 2 nmol/L — the highest PS affinity reported among known phospholipid-binding proteins [60, 61]. The high PS-binding affinity on PS-exposing cells, such as activated platelets and apoptotic cells, enables Annexin A5 to competitively shield PS from coagulation factor complexes, thus exerting potent anticoagulant and anti-inflammatory effects by disrupting the assembly of the prothrombinase complex and inflammatory cell signaling [31, 32]. The AB loop and specific amino acid residues, particularly Trp185 in Domain III, are pivotal in modulating membrane-binding strength. Trp185 is normally buried within the protein core but becomes exposed upon calcium binding, thereby dramatically enhancing membrane affinity [62]. This calcium-dependent exposure and binding mechanism enable Annexin A5 to selectively target pathological cells exposing PS, such as thrombotic or ischemic tissues, while sparing healthy cells, thereby enhancing its suitability for targeted drug delivery applications.

Additionally, upon membrane binding, Annexin A5 assembles into ordered two-dimensional lattice structures through trimerization on the phospholipid bilayer. These lattices generate localized membrane tension, facilitating membrane bending and invagination, which leads to the formation of vesicles capable of internalizing Annexin A5 along with associated therapeutic agents [58]. Notably, this endocytic pathway is active in PS-exposing cardiomyocytes and tumor cells under stress but remains quiescent in healthy tissues, which grants Annexin A5 high specificity and reducing off-target effects. Lastly, Annexin A5 is efficiently degraded within lysosomes following internalization via the endosomal pathway, mitigating concerns about intracellular accumulation or long-term cytotoxicity. This efficient catabolism enhances its favorable safety profile in therapeutic applications, as any excess protein is promptly metabolized and eliminated via intrinsic degradation pathways [63].

In conclusion, the distinctive structural features of Annexin A5 — encompassing its conserved domain architecture, calcium-binding sites, key amino acid residues, self-assembling trimeric lattice formation, and receptor-independent internalization — collectively underpin its anticoagulant, anti-inflammatory, and drug delivery functions, accompanied by a favorable safety profile conducive to clinical translation.

## 4. Multidimensional therapeutic potential of Annexin A5 in ischemic stroke

### 4.1 Antiplatelet and anticoagulant effects of Annexin A5 in ischemic stroke

Ischemic stroke is characterized by a sudden interruption of cerebral blood flow that leads to a series of pathophysiological events leading to neuronal damage and cerebral infarction [64]. Initially, platelets are activated by endothelial injury caused by ischemia and hypoxia, and PS on the outer membrane of activated platelets is exposed. This PS exposure further exponentially amplifies platelet activation, and subsequently activates thrombin, resulting in thromboembolism [65, 66]. Thrombosis and microvascular occlusion are important events in the early pathological stage of ischemic stroke. At present, anticoagulant therapy reduces ischemic stroke recurrence and deep vein thrombosis; However, some studies have pointed out that the use of some anticoagulants can increase the risk of bleeding [67]. At the same time, antiplatelet drugs are not satisfactory [68]. For example, as an anti-platelet aggregation drug, Aspirin is an effective drug to prevent ischemic stroke recurrence after the onset of ischemic stroke [69]. However, studies have shown that long-term use of aspirin as an antiplatelet drug increases the risk of gastrointestinal bleeding [70].

In the process of platelet activation, the externalization of PS provides a procoagulant surface that accelerates thrombin generation, promotes platelet aggregation, and stabilizes fibrin-rich thrombi, thereby aggravating cerebral ischemia and expanding infarct size [71]. Consequently, targeting PS-exposing cells within the ischemic microenvironment emerges as a promising therapeutic strategy to mitigate thrombotic burden, restore microcirculatory patency, and preserve neuronal viability. As a human-derived protein, Annexin A5 exhibits a high affinity for PS-positive membranes, positioning it as a potential agent for interrupting PS-mediated prothrombotic processes. Annexin A5 exhibits both anticoagulant and antiplatelet properties, possesses a short in vivo half-life, and can be rapidly metabolized after drug action, thereby reducing potential side effects and enhancing treatment safety. Annexin A5 is widely present in endothelial cells and platelets and is released in response to tissue injury [72]. Annexin A5 can bind to the exposed PS on the surface of platelets and block the reaction of PS with other procoagulant factors, thereby inhibiting the occurrence of blood coagulation [73, 74]. Additionally, Annexin A5 binds to glycosphingolipid (sulfatide) on the platelet surface, inhibiting the activation of coagulation factor XII [75]. In addition, Annexin A5 can inhibit endothelial cell-mediated thrombin formation and act as an anticoagulant [76] (Fig. 2.)

According to the properties of Annexin A5, Kuypers et al. [77] engineered a homodimeric variant of Annexin A5, termed Diannexin V (DAV), which demonstrated enhanced PS-binding affinity and a prolonged half-life of 6.5 hours. After collecting blood samples from humans and mice, the authors confirmed by flow cytometry that DAV bound more strongly to red blood cells exposed to PS than Annexin A5 monomer. In addition, DAV showed excellent antithrombotic activity in Wessler rat model. These results suggest that DAV has superior targeting ability to procoagulant surfaces and potential as an antithrombotic agent. Building on this PS-targeting capability, Tait et al. [78] used human blood-derived platelet-enriched plasma to construct clots and prepared chimeric proteins containing full-length or truncated pro-urokinase fused with Annexin V in the E. coli expression system. Using membrane binding assay, plasminogen activation assay and clot lysis assay in vitro, they found that these chimeras retained the PS binding ability of Annexin V, the enzymatic activity of urokinase and the thrombolytic function. This suggests that Annexin V can be used as a targeting component to effectively direct pro-urokinase to platelet-associated thrombi, which provides the feasibility for the development of new targeted thrombolytic agents. Further extending the functional potential of Annexin A5-based therapeutics, Jing et al. [79] expressed and purified a recombinant fusion protein consisting of the snake venom peptide echistatin and Annexin A5 (r-EchAV) in E. coli. Functional assays including PS-binding, plasma clotting, platelet aggregation, and flow cytometry demonstrated that r-EchAV bound to PS in a calcium-dependent manner, competitively inhibited fibrinogen binding to the platelet αIIbβ3 receptor, significantly prolonged clotting time, and suppressed ADP-induced platelet aggregation. In a mouse model, r-EchAV also dose-dependently prolonged bleeding time. This suggests that r-EchAV exerts dual anticoagulant and antithrombotic effects by simultaneously targeting PS and αIIbβ3, representing a potential novel therapeutic strategy against thrombosis.

Collectively, these findings highlight that PS exposure represents a critical therapeutic target in ischemic stroke pathophysiology, and Annexin A5-based compounds, by selectively binding PS-positive membranes, offer a promising strategy to disrupt platelet-driven thrombus propagation, alleviate microvascular obstruction, and potentially improve clinical outcomes in ischemic stroke management.

**Figure 2.**
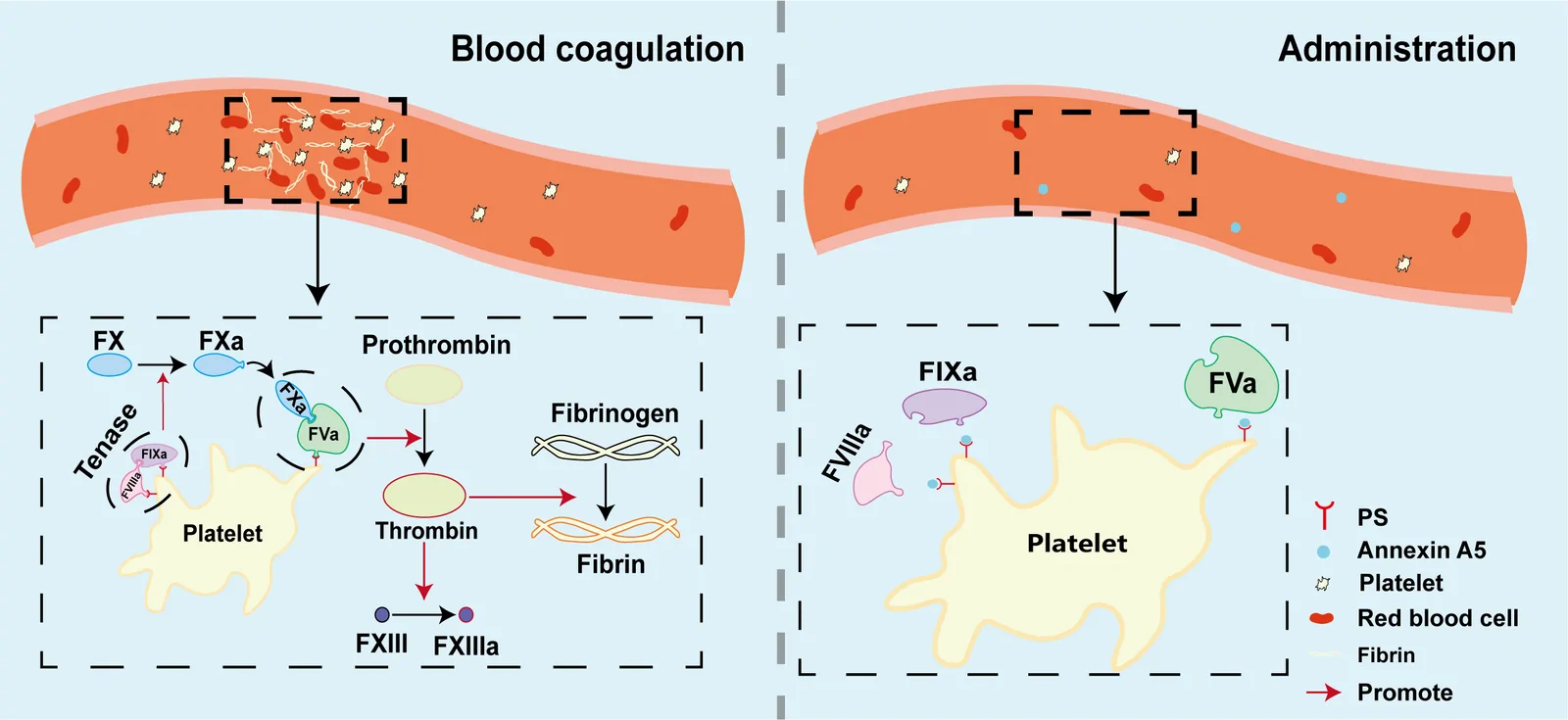
**Anticoagulant mechanism of Annexin A5**. When platelets are activated, thrombin activates factor FVa, which subsequently facilitates the formation of the Tenase complex by combining with hemophilia factor FVIIIa and activator FIXa. This complex promotes the conversion of FX to FXa. FXa then binds with FVa to form the prothrombinase complex, which catalyzes the transformation of prothrombin into thrombin, thereby enabling the extensive production of thrombin. PS enhances large-scale thrombin generation by interacting with both the Tenase complex and the prothrombinase complex. Annexin A5 will bind to PS to occupy the site and inhibit thrombin cascade activation as well as fibrin activation.

### 4.2 Anti-inflammatory effect of Annexin A5 in ischemic stroke.

In the pathological process of ischemic stroke, inflammatory responses play a critical role in exacerbating cerebral injury and impairing vascular function [80-82]. Ischemia-induced endothelial dysfunction disrupts the integrity of the BBB, further increasing vascular permeability and the release of pro-inflammatory mediators [31]. At the molecular level, several key inflammatory mediators contribute to this pathology. Damage-associated molecular patterns (DAMPs) such as HMGB1 and lipopolysaccharide (LPS) engage Toll-like receptor 4 (TLR4), activating the NF-κB axis and upregulating pro-inflammatory cytokines [83]. Additionally, macrophage polarization toward the pro-inflammatory M1 phenotype involves PKM2-mediated glycolytic reprogramming and accompanying STAT1/ NF-κB activation [84, 85]. Downstream cyclooxygenase-2 (COX-2) expression and leukotriene B4 (LTB4) synthesis via 5-LOX further amplify inflammatory lipid signaling [86]. Low-density lipoprotein (LDL), particularly in its oxidized form (OxLDL), has been shown to aggravate endothelial injury and promote vascular inflammation by inducing the expression of adhesion molecules such as ICAM-1 and VCAM-1 on endothelial cells [87]. This facilitates the recruitment of leukocytes and macrophages into the ischemic brain parenchyma, where these immune cells contribute to secondary neuronal injury through the production of reactive oxygen species, proteases, and cytokines. The interplay between vascular dysfunction, endothelial activation, LDL-induced oxidative stress, and immune cell recruitment forms a vicious cycle that accelerates tissue damage and worsens neurological outcomes in ischemic stroke [88].

Recombinant human Annexin A5 has demonstrated efficacy in preventing endothelial inflammation induced by LPS-activated platelets and micro-vesicles. In a rat MCAO model of focal cerebral ischemia, this effect is mediated through its ability to significantly reduce TNF-α and adhesion molecule expression, restore endothelial barrier integrity, and inhibit monocyte and platelet adhesion processes that are dependent on PS binding [89]. In addition to its vascular effects, Annexin A5 plays a key role in modulating immune responses. In murine models of sepsis and endotoxemia, Annexin A5 administration attenuated HMGB1-driven cytokine release and improved survival, emphasizing its function in suppressing DAMP/TLR4-mediated NF-κB signaling [90]. This anti-inflammatory effect is further supported in the context of vascular injury, where Annexin A5 therapy reduced leukocyte and platelet adhesion, vascular remodeling, and inflammation. Additionally, in a model of nonalcoholic steatohepatitis (NASH), Annexin A5 directly interacted with PKM2, inhibiting its phosphorylation at Y105, which led to a shift in macrophages from an M1 to M2 phenotype. This shift suppressed STAT1/NF-κB signaling pathways, thereby reducing the expression of pro-inflammatory cytokines such as IL-1β, IL-6, and TNF-α [91]. Moreover, in human prostate cancer cell lines, Baek et al. demonstrated that Annexin A5 also downregulates COX-2 expression in both endothelial and immune cells, providing an additional mechanism for controlling eicosanoid-mediated inflammation [92]. By binding PS-exposed micro-vesicles, Annexin A5 prevents interaction with endothelial and immune cells, thus broadly limiting PS-mediated pro-inflammatory and procoagulant signaling [93].

Additional evidence suggests that Annexin A5 has therapeutic potential to improve vascular inflammation. Atherosclerosis (AS) is a chronic inflammatory vascular disease, which is one of the causes of ischemic stroke [94]. Plaque rupture secondary to AS accounts for a significant proportion of cerebrovascular thromboembolic events [95]. A key molecular mediator implicated in AS pathology is lysophosphatidylcholine (LPC), a pro-inflammatory phospholipid generated through the oxidative and enzymatic modification of LDL [96]. LPC disrupts endothelial homeostasis, promotes leukocyte recruitment, and accelerates local vascular inflammation, thereby contributing to plaque destabilization. Moreover, oxidized LDL (OxLDL) exacerbates these processes by enhancing macrophage activation and foam cell formation within atherosclerotic lesions [87]. Recent evidence highlights the therapeutic potential of Annexin A5 in this context. Systemic injection of Annexin A5 in mice with damaged vessels can effectively remodel vasculature and improve vascular function, suggesting that Annexin A5 may play a potential role in the treatment of AS [97]. In addition, AS leads to an inflammatory state and plaque rupture is a major cause of cardiovascular disease. In the co-culture of human endothelial cells and macrophages, Annexin A5 can reduce the proinflammatory effects of OxLDL and LPC, inhibit macrophage binding and uptake of OxLDL, and play an important protective role in the occurrence of AS and plaque rupture [98]. These findings collectively position Annexin A5 as a promising candidate for targeting lipid-driven vascular inflammation and reducing ischemic stroke risk in the setting of advanced atherosclerotic disease.

Furthermore, in a mouse model mimicking percutaneous coronary intervention (PCI), Annexin A5 was found to inhibit the recruitment of white blood cells and macrophages in a dose-dependent manner, thereby reducing inflammation after vascular injury [99]. Gao et al. [100] established a traumatic brain injury (TBI) model in mice and administered recombinant human Annexin A5 (50 μg/kg) via tail vein injection. Behavioral assessments showed improved neurological and motor function following Annexin A5 treatment. Western blot analysis revealed decreased high mobility group box-1 (HMGB1) expression and upregulation of heme oxygenase-1 (HO-1), suggesting that Annexin A5 exerts neuroprotective effects by modulating HMGB1 signaling and the HO-1 antioxidant system, thereby alleviating neuroinflammation and oxidative stress. In a separate study, De Jong et al. [101] used hypercholesterolemic ApoE*3-Leiden transgenic mice to investigate myocardial ischemia-reperfusion (MI-R) injury. Annexin A5 treatment significantly reduced myocardial infarct size, cardiac end-diastolic volume, proliferative macrophage infiltration, and interleukin-6 levels in the infarct and peri-infarct regions. These findings indicate that Annexin A5 attenuates post-ischemic inflammation and improves ventricular remodeling and cardiac function following MI-R injury. Moreover, this anti-inflammatory effect on myocardial tissue suggests a potential role for Annexin A5 in preventing cardiogenic ischemic stroke.

### 4.3 Anti-apoptotic and neuroprotective effects of Annexin A5.

Apoptosis represents a primary contributor to delayed neuronal injury, profoundly influencing both infarct progression and long-term neurological outcome [102, 103]. One of the earliest cellular changes during apoptosis is the disruption of plasma membrane phospholipid asymmetry, leading to the externalization of PS from the inner to the outer leaflet of the cell membrane [104]. PS exposure is a hallmark of early apoptosis. It also functions as an “eat me” signal, facilitating recognition and phagocytic clearance of stressed neurons by microglia and macrophages, contributing to progressive neuronal loss in ischemic regions [105, 106]. In addition, central to the intrinsic pathway, Bcl-2 family proteins, including Bcl-2 and Bax, control mitochondrial outer membrane permeabilization (MOMP) and subsequent cytochrome c release, initiating downstream caspase activation. Among them, in the apoptotic signaling pathway, caspase-3 is an important downstream apoptotic execution molecule, driving DNA fragmentation and neuronal death [107]. Simultaneously, dysregulated intracellular calcium homeostasis, mediated largely by voltage-dependent anion channel 1 (VDAC1), promotes mitochondrial dysfunction and exacerbates apoptotic signaling [108, 109]. These molecular—including Bcl-2/Bax, VDAC1, calcium imbalance and caspase activation—collectively represent attractive therapeutic targets for preserving penumbral neuronal viability [103, 110, 111].

**Figure 3.**
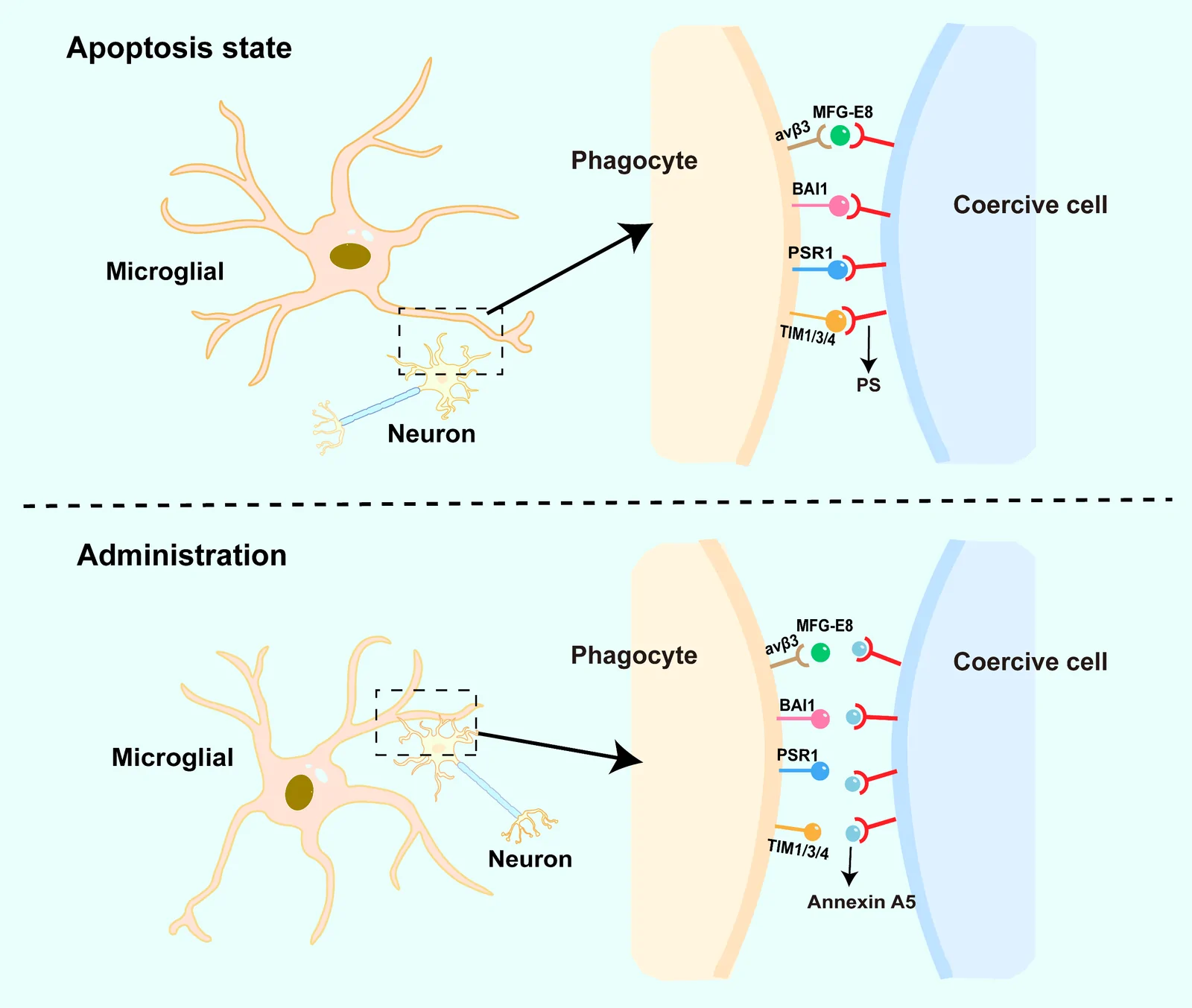
**Mechanism of Annexin A5 anti-apoptosis effect**. Microglia recognize PS exposed on damaged cells via factors such as the avβ3/MFG-E8 complex, BAI1, PSR1, and TIM1/3/4 present on their own membranes, thereby initiating subsequent cell death pathways. Annexin A5 binds to PS on the surface of damaged cells, thereby inhibiting microglial recognition.

Annexin A5 effectively masks these "eat me" signals by binding to PS exposed on the surface of apoptotic neurons, competitively inhibiting the interaction between PS and PS recognition receptors on phagocytes. Tang et al. [112] recently developed an Annexin A5 dimer using molecular docking technology. Based on the mouse MCAO model and primary astrocyte-microglia co-culture experiments, Annexin A5 dimers have an enhanced affinity for PS-exposed astrocytes and neurons compared to Annexin A5 monomers. This increased affinity enables Annexin A5 dimers to more effectively inhibit macrophage-mediated phagocytosis and in turn prevent astrocyte-associated BBB disruption, thereby contributing to the reduction of brain damage after ischemic stroke. Exogenous Annexin A5 administration reduces neuronal apoptosis and preserves neuronal populations in ischemic models. Takei et al. [33] found that Annexin A5 mRNA is expressed in non-neuronal cells of the central nervous system, and used the primary cortical neurons of embryonic rats to test the viability of neurons. Different concentrations of recombinant Annexin A5 were added to the culture medium on day 1 and day 3. The number of surviving neurons was maximized when recombinant Annexin A5 was administered at a concentration of about 30 ng/ml. When Annexin A5 antibody was added to the culture medium, the neurotrophic activity of Annexin A5 was inhibited. Annexin A5 protein from non-neurons can promote the survival of fetal rat cortical neurons cultured in vitro and has a neuroprotective effect on neurons, suggesting that Annexin A5 may be a potential therapeutic agent for long-term recovery after ischemic stroke, its role in neuron-non-neuronal interactions may represent a beneficial therapeutic approach in ischemic stroke. Furthermore, studies have provided strong evidence for the direct brain-protective effect of Annexin A5. In rodent models of stroke, hu et al. [25] found that Annexin A5 selectively accumulates in ischemic brain areas, reduces infarct size and improves neurological function after cerebral ischemia. This protective effect was abolished when Annexin A5 antibody was used. These findings suggest a neuroprotective role of Annexin A5 in ischemic stroke. Several lines of evidence suggest that Annexin A5 also influences changes in a number of apoptosis-related factors, thereby exerting a protective effect. Moreover, in the human CEM T cell line, Annexin A5 modulates intracellular calcium dynamics by inducing a controlled Ca²^+^ influx, which stabilizes calcium homeostasis and attenuates caspase-3 activation under apoptotic stress [113]. In addition, Annexin A5 has been identified as a direct regulator of VDAC1-mediated mitochondrial calcium homeostasis in endothelial cells extracted from Annexin A5-KO mice. By binding to mitochondrial membranes, it modulates VDAC1 oligomerization, maintains mitochondrial membrane potential, and prevents cytochrome c release in cells exposed to apoptotic stimuli [114]. Furthermore, a cell-free fat extract-derived form of Annexin A5 demonstrated anti-apoptotic effects by regulating macrophage polarization and reducing chondrocyte apoptosis, emphasizing its broader cytoprotective potential in inflammation-associated degenerative diseases [115]. The human Annexin A5 homodimer, Diannexin, exhibits potent anti-apoptotic effects. Shen et al. [116] found that Diannexin treatment reduced the expression of TNF-α, IL-1β, and IP-10, reduced the expression of P-selectin and endothelial ICAM-1, and alleviated the infiltration of macrophages and CD4 cells in a rat liver transplantation model compared with a control group without treatment. In addition, Diannexin increased the expression of HO-1/Bcl-2/Bcl-xl and decreased the expression of Caspase-3/TUNEL+ apoptotic cells.

In summary, current evidence positions Annexin A5 as a versatile cytoprotective agent capable of targeting multiple key apoptotic regulators—including Bcl-2/Bax, VDAC1, calcium dysregulation and caspase activation. Its ability to modulate these effectors offers a promising therapeutic strategy for limiting apoptotic neuronal loss and improving functional outcomes in ischemic stroke (Table 1).

**Table 1:** Summary of the mechanisms and biological relevance of Annexin A5 in the research model related to ischemic stroke.

Research object	Target	Mechanisms	Effect	Significance	Refs.
Clinic/Human	Brain damage Cells	—	AnxA5 recognizes neurons damaged by ischemia	Detection of Brain damage	[117]
Clinic/Human	Brain damage Cells	—	AnxA5 imaging can assess ischemic brain injury	Detection of Brain damage	[118]
Basic/Mouse	Brain damage Cells	—	The fluorescence intensity was higher on the injured side	Detection of Brain damage	[119]
Basic/In vitro	Apoptotic neurons	High affinity of AnxA5 for PS	Apoptotic neurons are labeled	Detection of apoptotic neurons	[120]
Clinic/Human	Antiphospholipid monoclonal antibodies	AnxA5 two-dimensional crystallization hindered the coagulation reaction	Antiphospholipid monoclonal antibodies reduce the anticoagulant activity of AnxA5	Anticoagulant	[121]
Basic/Mouse + in vitro	Bone marrow macrophages	IL-6 down-regulate	Left ventricular remodeling and function were improved	Anti-inflammatory	[101]
Basic/Mouse + in vitro	Gut tissue	Activate Nrf2/HO-1, inhibits HMGB1	Improve intestinal mucosal injury induced by traumatic brain injury	Anti-inflammatory	[122]
Basic/In vitro	Leydig and Sertoli cells	Activate ERK/Nrf2 pathway	Oxidative stress damage was reduced in Leydig and Sertoli cells	Anti-inflammatory	[123]
Basic/Mouse + in vitro	Platelets and endothelial cells	Inhibit the expression of inflammatory factors and adhesion molecules	The survival rate of mice increased	Anti-inflammatory	[89]
Basic/Mouse	Phagocytic	IL-10 up-regulate	The immune response of AnxA5-KO mice to allogeneic necrotic cells was weakened	Anti-inflammatory	[124]
Basic/Rabbit+Mouse	Ischemic myocardium	High affinity of AnxA5 for PS	AnxA5 targets PS exposed to cardiomyocytes	Damage detection	[125]
Basic/Mouse	Phagocytic	Inhibit the infiltration and activation of macrophages	Plaque formation in the atherosclerotic model is reduced	Anti-apoptosis	[126]
Basic/In vitro	SH-SY5Y cells	ANXA5 inhibits ROS accumulation and MMP loss	The viability of SH-SY5Y cells increased	Anti-apoptosis	[127]
Basic/Rat + in vitro	Renal cell membrane	AnxA5 forms a two-dimensional network structure	Maintenance of renal cell membrane integrity	Anti-apoptosis	[128]
Basic+Clinic/Mouse+Hum-an	—	AnxA5 in the lungs is transferred to the brain	Reduce infarct volume and improve neurological function	Neuroprotection	[25]
Basic/Mouse	Platelets	High affinity of AnxA5 for PS	Significant thrombolytic and neurological function improvement	Construction of targeted vector	[129]
Basic/Mouse	Sinusoidal endothelial cells	Inhibit MIP-2, ICAM-1 and VCAM	Interrupt secondary microcirculation inflammation and coagulation	Anti-inflammatory	[130]
Basic/Rat	Endothelial cells	Inhibits the binding of leukocytes to endothelial cells	Blood flow is increased and vascular permeability is decreased	Anti-inflammatory	[131]
Basic/Rat	Endothelial cells	Inhibit TNF-α'IL-1β'IP-10, Upregulate HO-1/Bcl-2/Bcl-xl	Improve cold ischemia-reperfusion injury	Anti-inflammatory, Anti-apoptosis	[116]
Basic/Mouse	Renal ischemic tissue	Inhibition of leukocyte and central granulocyte infiltration	Improve renal ischemia-reperfusion injury	Anti-inflammatory	[132]
Basic/Rabbit	Brain damage Cells	—	AnxA5 labeled the injured cells	Detection of Brain damage	[133]
Basic/Mouse	Phagocytic	Inhibit TLR4/NF-κB/NLRP3	Improve myocardial ischemia-reperfusion injury	Anti-inflammatory	[134]
Basic/Rat	—	Inhibit cytokeratin 18 and IL-6	Improve ischemia-reperfusion injury in lung transplantation	Anti-inflammatory	[135]
Basic/Mouse	Astrocytes/Microglia	Inhibition of microglial phagocytosis	Protect the integrity of the blood-brain barrier after cerebral ischemia	Anti-apoptosis, BBB protection	[112]
Basic/Mouse + in vitro	Dermal fibroblasts	Inhibit the phosphorylation of Smad2	Reduce dermal thickness and collagen deposition	anti-fibrotic, anti-inflammatory	[136]

Abbreviations: AnxA5: Annexin A5; Nrf2: Nuclear factor E2-related factor 2; HO-1: hemeoxygenase-1; HMGB1: high mobility group box 1; IRI: Ischemia-reperfusion injury

## 5. Application of Annexin A5 in the diagnosis and treatment of ischemic stroke

### 5.1 Annexin A5 can assist in the localization of target lesions

Following the occurrence of ischemic stroke, accurate assessment of the location and size of the infarction is crucial. Currently, most patients are diagnosed using neuroimaging modalities. Annexin A5 has emerged as a promising molecular probe for monitoring ischemic stroke. Lee et al. [117] investigated the clinical significance of anti-Annexin A5 antibodies (aAV) in patients with acute cerebral ischemia using enzyme-linked immunosorbent assays to detect aAV protein levels in patient and control groups. The results demonstrated a higher proportion of aAV-positive subjects in the patient group. In the same age group (35-75 years), the positive rate of aAV in the patient group remained significantly higher than in the control group, suggesting that the detection rate of aAV is elevated in patients with acute ischemic stroke and should be considered a potential related factor.

Annexin A5 can be conjugated with various labeled signaling molecules for qualitative or quantitative evaluation. Mari et al. [118] used technetium-99m, a nuclear isomer of technetium-99, to label Annexin A5, creating technetium-99m-Annexin A5, and evaluated whether it could noninvasively monitor neuronal damage after focal middle cerebral artery occlusion/reperfusion injury. The results indicated a higher uptake of Annexin A5 was observed in damaged brain tissue regions following MCA occlusion/reperfusion injury, suggesting that Annexin A5-based tracers can effectively monitor neuronal damage following focal MCA occlusion/reperfusion injury. Furthermore, Blankenberg et al. [119] prepared technetium-99m hydrazine nicotinamide-labeled Annexin A5 (99mTc-HYNIC-Annexin A5) to determine its utility in evaluating ischemic injury in patients with acute ischemic stroke and treatment efficacy in animal models of focal ischemic injury. Clinical experiments showed that the area of 99mTc-HYNIC-Annexin A5 uptake in patients corresponded with brain damage identified by MRI. Animal experiments revealed reduced uptake rates of 99mTc-HYNIC-Annexin A5 in the midbrain of the treatment group, decreased numbers of caspase-8-stained neurons, and reduced cerebral infarction areas. These findings suggest that Annexin A5 imaging can be employed to evaluate the therapeutic effects of ischemic brain injury. Thus, Annexin A5-based imaging holds promise for enhancing diagnostic precision and monitoring therapeutic efficacy in cerebrovascular disease.

### 5.2 Annexin A5 can be involved in the construction of targeting vectors in ischemic stroke

A major challenge that continues to impede the successful clinical translation and broad application of therapeutic agents is the difficulty in achieving precise and efficient drug delivery to the intended target sites within the human body [120]. This issue is especially critical in neurological diseases, where the anatomical and physiological features of the CNS present formidable barriers to drug delivery. One of the most significant obstacles to targeted drug delivery in the brain is the presence of the BBB [121, 122], a highly selective, semipermeable interface that tightly regulates the exchange of molecules between the circulatory system and neural tissue. A large number of promising drugs, including neuroprotective agents, chemotherapeutic compounds, and disease-modifying biologics, fail to achieve therapeutic concentrations in the brain, thereby limiting their clinical efficacy. [121] Overcoming the BBB has thus become a major focus of ongoing research in the fields of drug delivery, nanomedicine, and neuropharmacology. Various strategies have been explored to enhance drug penetration across the BBB, including the use of nanoparticle-based carriers, liposomes, exosomes, receptor-mediated transcytosis, ultrasound-mediated drug delivery, and chemical modifications of drug molecules to improve their lipophilicity and transport efficiency [123-125]. Despite these advances, the development of a universally effective and clinically applicable method for precise and efficient drug delivery to the brain remains an unresolved issue. Annexin A5 has a high affinity for PS, a phospholipid that is exposed to the extracellular membrane when cells are dying [126-128]. Some researchers have noticed this targeting property of Annexin A5 to dying cells, and have used it to construct a drug carrier that can target ischemic lesions to treat ischemic stroke [112]. Quan et al. [129] developed an Annexin A5 Inserted Platelet Fusogenic Liposome-tissue plasminogen activator (APLT-PA). It was found that mice injected with APLT-PA had significantly reduced infarct volume at 72 hours after administration and infarct volume at 7 days after administration, as well as a significant reduction in BBB permeability, the result suggests the feasibility of using Annexin A5 as a targeted vector to treat ischemic stroke. This study provides a novel and safe platelet-mimicking nanomedicine for precise thrombolytic therapy of ischemic stroke and proposes a new theoretical basis for the design and development of cell-mimetic nanomedicine for a variety of biomedical applications. The ability of Annexin A5 to target lesions can greatly reduce the possible or undetected side effects of common drugs on normal tissues, reduce the excessive dose and cost of drug use caused by fluid dilution, and increase the safety and effectiveness of treatment.

**Figure 4.**
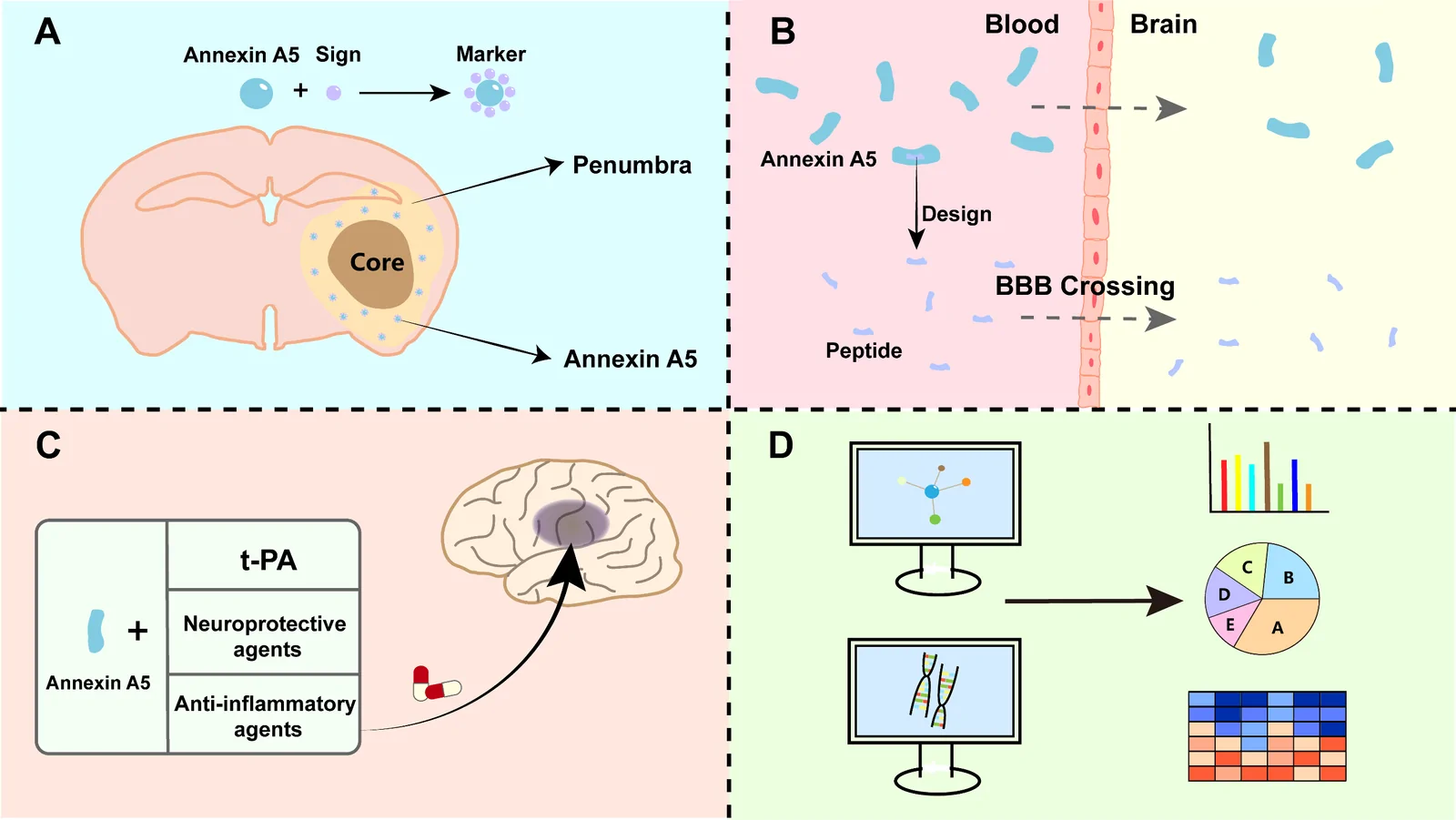
**Novel applications of Annexin A5**. (**A**) Annexin A5 was conjugated with signal molecules to develop a molecular marker specifically targeting the ischemic penumbra, enabling its visualization and precise localization. (**B**) Based on the structural properties of Annexin A5, functionally similar small molecular peptides were designed to enhance the ability to cross the blood-brain barrier. (**C**) Annexin A5 was linked with various therapeutic or neuroprotective agents, leveraging its PS-targeting capability to facilitate targeted drug delivery to the affected lesion site. (**D**) To investigate the more intricate functions and underlying mechanisms of Annexin A5, a comprehensive multi-omics analysis was conducted.

## 6. Limitations of Annexin A5 in ischemic stroke

Although preclinical studies have shown that Annexin A5 reduces infarct volume and improves neurological outcomes in rodent models of focal cerebral ischemia [25, 130], these findings remain preliminary. Clinical experience with recombinant Annexin A5 is limited to early-phase pharmacokinetic and safety studies in non-stroke settings, such as COVID-19 [131, 132]. There are not randomized trials to confirm its efficacy in human ischemic stroke at present.

From a mechanistic perspective, PS externalization after cerebral ischemia displays spatial and temporal heterogeneity influenced by cell type, reperfusion status, and injury severity [104], complicating the targeting of PS, therapeutic window selection, and biomarker development. Given Annexin A5’s high affinity for PS and its potential interference with the phospholipid-dependent coagulation cascade [133], administration may carry risks of off-target effects, including hemostatic and inflammatory disturbances. Although studies in other diseases reported no acute coagulation changes, these excluded high-risk populations [131, 132]. It is still necessary to explore these changes in ischemic stroke.

Furthermore, significant uncertainties remain regarding the delivery, bioavailability, and target engagement of Annexin A5 within the ischemic penumbra [134]. For instance, Annexin A5-based imaging and tracer studies are limited by rapid renal clearance and technical challenges in labeling and detection [119]. These limitations hinder the accurate determination of effective concentrations and duration of Annexin A5 exposure in injured brain tissue.

Another critical issue pertains to the long-term immunogenicity, consistency in manufacturing, and regulatory considerations for Annexin A5 therapy. Parameters, including antidrug antibody formation, batch-to-batch stability, and the requirement for indication-specific safety data, have not been systematically evaluated in clinical cohorts of ischemic stroke patients. Consequently, the overall safety profile of Annexin A5 must be further validated through extensive clinical investigation.

## 7. Perspective and future directions

### 7.1 Can Annexin A5 become a new key to unlock antithrombotic lock^+^

Annexin A5 inhibits coagulation and platelet aggregation by binding PS on activated platelets and endothelial cells, blocking prothrombinase and tenase complex formation [135]. However, Annexin A5 large molecular weight limits BBB penetration, and short half-life reduces therapeutic duration. Additionally, its anticoagulant effect is confined to PS-positive surfaces without direct inhibition of coagulation factors in plasma. To improve this, optimized low-molecular-weight derivatives or PS-binding peptides with better BBB permeability and longer half-life should be developed. Combination with thrombolytics or antiplatelet agents may also enhance recanalization while reducing bleeding risk.

### 7.2 Can Annexin A5 become a new fire extinguisher in ischemic stroke inflammation^+^

Annexin A5 suppresses neuroinflammation by binding PS-exposing apoptotic cells and micro-vesicles, reducing leukocyte adhesion and inflammatory signaling [89]. However, its precise intracellular anti-inflammatory mechanisms remain incompletely defined in the ischemic stroke, particularly concerning pathways such as NF-κB activation and inflammasome assembly. Additionally, its limited tissue retention diminishes prolonged local anti-inflammatory effects. Future research should clarify the downstream molecular signaling regulated by Annexin A5 in microglia and endothelial cells, using transcriptomic and proteomic approaches, and identify strategies to enhance its local anti-inflammatory persistence in damaged brain tissue.

### 7.3 Can Annexin A5 protect apoptotic cells^+^

Annexin A5 protects neurons in the ischemic penumbra by binding PS on apoptotic cells and preventing PS clearance by microglia [136]. However, its anti-apoptotic effect is limited to the PS shielding at the membrane level, and its regulation of the intrinsic neuronal apoptosis pathway and the targets for intervention should be further explored. To further elucidate this function of Annexin A5, studies should thoroughly analyze the potential intracellular role of Annexin A5 in neurons under ischemic conditions, especially its effect on the apoptotic signaling cascade, to determine whether it can provide direct neuroprotective effects beyond PS binding.

### 7.4 Can Annexin A5 become a radar for accurately targeting ischemic penumbra lesions^+^

Annexin A5 selectively localizes to brain lesions by targeting PS-exposing apoptotic cells, enabling molecular imaging of infarcted areas [137]. However, current radiolabeled Annexin A5 agents show limited brain uptake and imaging resolution, which impairs precise delineation of penumbra-core boundaries in vivo, especially during early stroke phases. Future directions should focus on developing second-generation radiotracers or Annexin A5 derivatives with improved signal-to-noise ratio and optimized pharmacokinetics for high-resolution lesion imaging.

### 7.5 Can Annexin A5 become a transport vehicle for targeting lesions^+^

Annexin A5 can serve as a targeting ligand for delivering therapeutics to PS-exposing cells and vesicles in lesions [138]. However, its use as a drug delivery platform faces limitations in drug-loading capacity, stability of conjugates, and potential off-target binding in peripheral tissues, reducing targeting specificity. Therefore, future studies should refine Annexin A5-based carrier systems by improving their loading efficiency, conjugate stability, and PS-binding selectivity, ensuring precise and effective drug delivery exclusively to pathological sites.

## 8. Summary

Annexin A5 has garnered increasing attention as a multifunctional endogenous molecule capable of concurrently modulating several critical pathophysiological cascades implicated in ischemic stroke progression. Through high-affinity binding to PS exposed on activated platelets, injured endothelial cells, and apoptotic neurons, Annexin A5 targeting property not only interrupts thrombogenic amplification and inflammatory cell recruitment but also preserves neuronal viability by inhibiting microglial efferocytosis of penumbral neurons, future studies should prioritize the systematic dissection of the downstream molecular signaling pathways modulated by Annexin A5 in ischemic brain injury. Additionally, the rational design and preclinical validation of multifunctional Annexin A5-based nanotherapeutics capable of co-delivering thrombolytic, anti-inflammatory, and neuroprotective agents represent a highly promising translational strategy. As cerebrovascular medicine continues to evolve toward network-based and specific patient intervention paradigms, Annexin A5 stands poised to serve as a pivotal molecular tool in next-generation ischemic stroke diagnostics, prognostics, and therapeutics. Its unique combination of endogenous origin, multi-target functionality, and expanding translational applications positions it as an attractive candidate for reshaping future cerebrovascular intervention strategies.
